# SUN2 exerts tumor suppressor functions by suppressing the Warburg effect in lung cancer

**DOI:** 10.1038/srep17940

**Published:** 2015-12-10

**Authors:** Xiao-bin Lv, Lijuan Liu, Chun Cheng, Bentong Yu, Longxin Xiong, Kaishun Hu, Jianjun Tang, Lei Zeng, Yi Sang

**Affiliations:** 1 Nanchang Key Laboratory of Cancer Pathogenesis and Translational Research, Center Laboratory, the Third Affiliated Hospital, Nanchang University, Nanchang, China; 2 Department of Pharmacy, Jiangxi cancer Hospital, Nanchang, China; 3 Department of cardiothoracic surgery, the First Affiliated Hospital, Nanchang University, Nanchang, China; 4 Guangdong Provincial Key Laboratory of Malignant Tumor Epigenetics and Gene Regulation, Medical Research Center, Sun Yat-Sen Memorial Hospital, Sun Yat-Sen University, Guangzhou, China

## Abstract

SUN2, a key component of LINC (linker of nucleoskeleton and cytoskeleton) complex located at the inner nuclear membrane, plays unknown role in lung cancer. We found that SUN2 expression was decreased in lung cancer tissue compared with paired normal tissues and that higher SUN2 levels predicted better overall survival and first progression survival. Overexpression of SUN2 inhibits cell proliferation, colony formation and migration in lung cancer, whereas knockdown of SUN2 promotes cell proliferation and migration. Additionally, SUN2 increases the sensitivity of lung cancer to cisplatin by inducing cell apoptosis. Mechanistically, we showed that SUN2 exerts its tumor suppressor functions by decreasing the expression of GLUT1 and LDHA to inhibit the Warburg effect. Finally, our results provided evidence that SIRT5 acts, at least partly, as a negative regulator of SUN2.Taken together, our findings indicate that SUN2 is a key component in lung cancer progression by inhibiting the Warburg effect and that the novel SIRT5/SUN2 axis may prove to be useful for the development of new strategies for treating the patients with lung cancer.

Lung cancer is the leading cause of cancer-related mortality worldwide^1^,^2^,^3^, being associated with an overall 5-year survival rate lower than 16%^4^,^5^,^6^.The dysregulation of oncogenes or tumor suppressor genes is tightly correlated with the initiation, progression and resistance to therapy of lung cancer^7^,^8^,^9^, all of which involve changes in the biological characteristics of cancer cells, including cell growth, apoptosis, migration, invasion, and metabolism. However, the molecular mechanisms of lung cancer pathogenesis are still poorly understood. Therefore, the identification of novel therapeutic targets, to develop less toxic therapies, as well as better predictive markers is urgently needed.

SUN2, a Sad1 and UNC84 domain protein, is located at the inner nuclear membrane, extending into the perinuclear space via its C-terminal SUN domain and interacting with the nuclear lamina through its nucleoplasmic N-terminal domain^10^,^11^,^12^. SUN2 is a member of the inner nuclear membrane LINC (linker of nucleoskeleton and cytoskeleton) complex, which maintains nuclear structure by connecting the nuclear lamina to the cytoskeleton^10^. Dysregulation of the LINC complex is associated with many human diseases, including cancers^10^,^11^,^13^. Hsieh TH *et al*. demonstrated, for the first time, that SUN2 plays a tumor suppressor role in miR-221/22-mediated malignant central nervous system embryonal tumors, in which SUN2 inhibits cell proliferation and tumor malignancy both *in vitro* and *in vivo*^14^. The mRNA and protein levels of SUN2 are decreased in breast tumor tissues compared with mammary epithelial tissues^15^. However, the role of SUN2 in lung cancer remains uncharacterized, and the mechanism by which SUN2 regulates cancer cell biological characteristics also needs to be addressed.

The majority of cancer cells exhibit elevated glucose uptake and lactate production, regardless of oxygen availability^16^,^17^, which has long been known as aerobic glycolysis, or the Warburg effect. The Warburg effect promotes cancer cell growth and evasion of apoptosis and can be targeted for cancer therapy^18^. It has recently been reported that loss of tumor suppressors or activation of oncogenes is involved in the Warburg effect. For example, the oncogenes AKT, c-Myc, and Ras promote the Warburg effect, whereas the tumor suppressors p53 and PTEN inhibit the Warburg effect in cancer cells^17^,^18^,^19^. However, the molecular mechanism underlying the Warburg effect in cancer cells is not fully understood.

In this study, we provide the first evidence that SUN2 plays a tumor suppressor role by suppressing the expression of GLUT1 and LDHA to inhibit the Warburg effect in lung cancer. We also show that the down-regulation of SUN2 is at least partly mediated by class III of the sirtuin family member SIRT5 in lung cancer. This novel SIRT5/SUN2 axis may be valuable for developing new strategies for treating patients with lung cancer.

## Results

### *SUN2* expression is decreased in lung cancer samples and higher *SUN2* levels predict a good outcome

**Figure 1:**
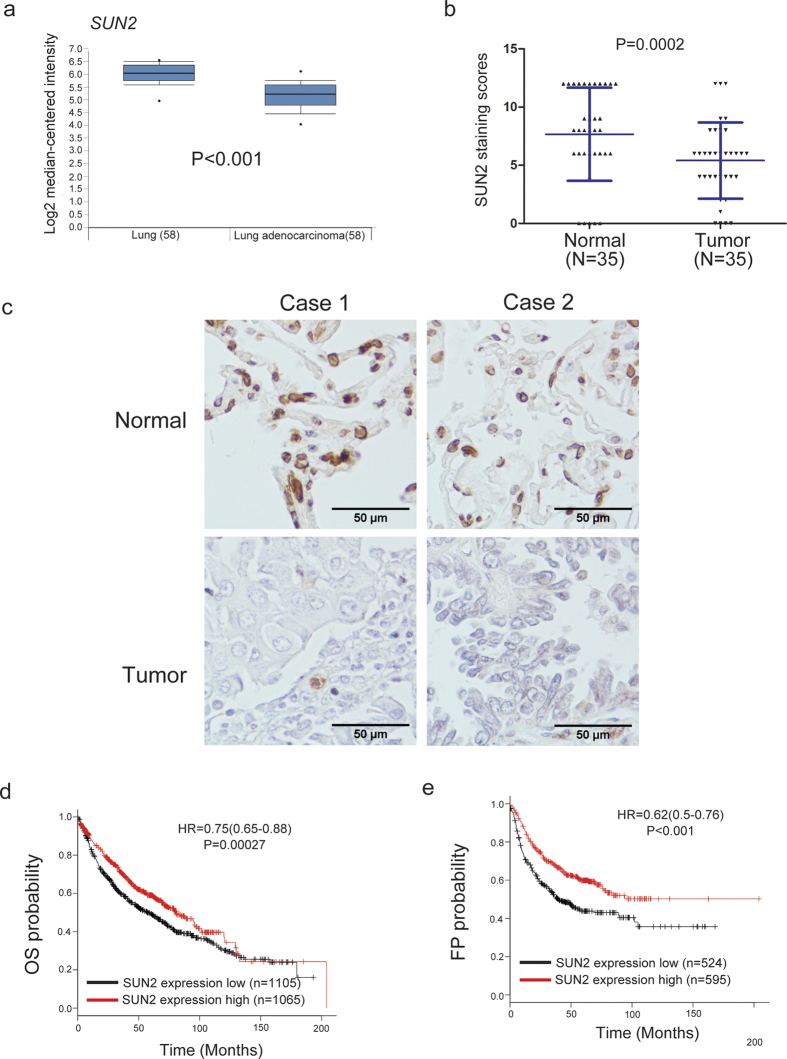
*SUN2* expression is decreased in lung cancer samples, and higher *SUN2* levels predict a good outcome. (**a**) Meta-analysis of *SUN2* mRNA levels in lung cancer samples from the Oncomine database (http://www.oncomine.org).Box plots showing the decreased expression of *SUN2* during tumorigenesis in lung adenocarcinoma datasets (Ref. 20). The y axis represents the log2 median-centered intensity (normalized expression). Shaded boxes represent the interquartile range (25^th^–75^th^ percentile). Whiskers represent the 10^th^–90^th^ percentile. The bars denote the median. The P value was calculated according to the raw data using Student’s t-test. (**b,c**) IHC of clinical lung cancer samples of both tumor and the paired normal tissues. Panel (**c**) presents representative images, and panel **b** illustrates the statistical results based on the Mann-Whitney test (P = 0.0002).The dots represent the scores, and the bars indicate the SD. Normal: normal lung tissues, n = 35; Tumor: lung cancer tissues, n = 35. Scale bars in **c**, 50 μm. (**d,e**)Lung cancer patients with low SUN2 expression exhibited significantly shorter overall survival (OS) and first-progression survival (FP) compared with those with high expression, P < 0.001.

To evaluate the expression level of *SUN2* in lung cancer, we analyzed its expression in lung cancer and normal lung tissues using the Oncomine database^20^. As shown in Fig. 1a, *SUN2* is significantly down-regulated in lung cancer tissues compared with the paired normal tissues (P < 0.01). However, SUN1, another Sad1 and UNC84 domain protein in the LINC complex, is not significantly differently expressed between lung cancer tissues and normal lung tissues (Supplementary Fig. S1). Interestingly, according to the Protein Atlas database, the protein expression level of SUN2 is decreased in 75% (9 out of 12) of lung cancer tissues (Supplementary Fig. S2). We also confirmed that SUN2 protein expression is significantly reduced in most cases of lung cancer through immunohistochemistry analysis (Fig. 1b,c).Moreover, Oncomine data mining revealed that *SUN2* is significantly down-regulated in other human cancers, such as cervical carcinoma, colorectal cancer, esophageal carcinoma and oral cavity squamous cell carcinoma^21^,^22^,^23^,^24^ (Supplementary Fig. S3), suggesting that SUN2 expression is closely related to cancer initiation or progression. Hence, we evaluated whether the *SUN2* mRNA level is clinically relevant. Based on the *SUN2* expression levels reported in a large public clinical microarray database of lung tumors from 2,170 patients^25^, lung cancer samples were subdivided into two groups, and the associated overall survival was analyzed. Individuals with low *SUN2* levels were observed to exhibit shorter overall survival (OS) than those with high levels (Fig. 1d).Furthermore, lower SUN2 mRNA expression levels are correlated with a shorter first progression (FP) survival than higher expression levels (Fig. 1e).These results indicate that SUN2 is down-regulated in lung cancer and that its high expression predicts a good outcome in lung cancer.

### SUN2 inhibits lung cancer cell proliferation and migration

**Figure 2:**
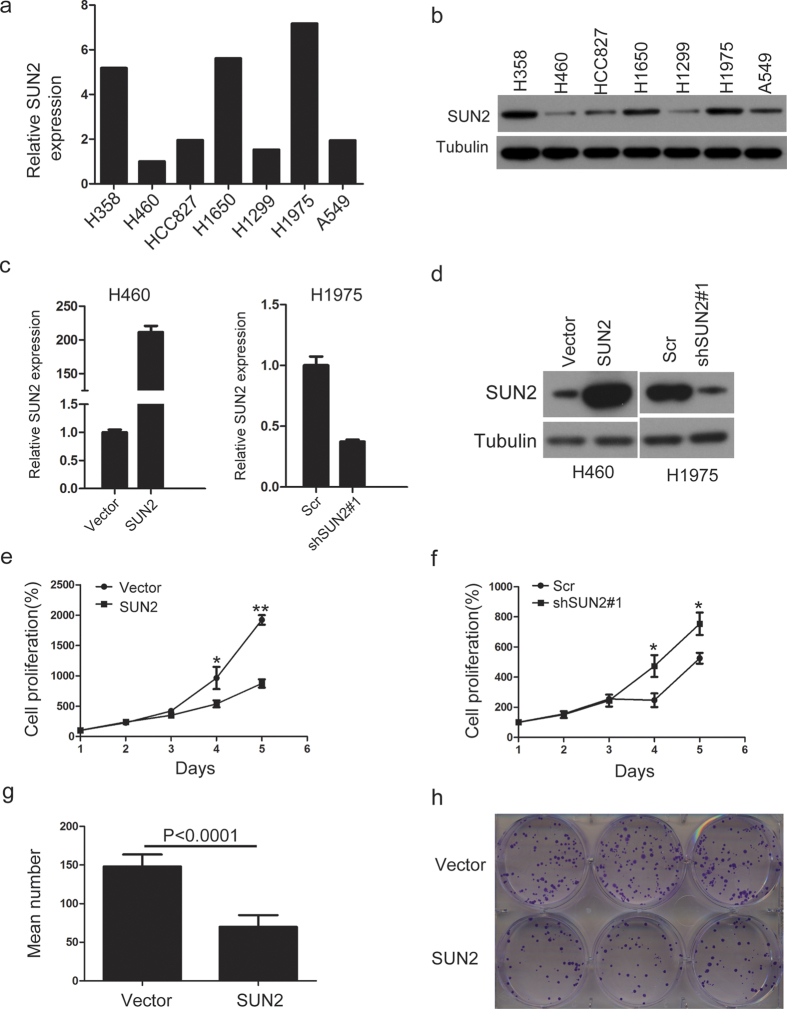
SUN2 inhibits lung cancer cell proliferation. (**a,b**) The mRNA and protein levels of SUN2 were determined in 7 lung cancer cell lines through qRT-PCR (**a**) and western blotting (**b**), respectively. GAPDH was used as an internal control in (**a**). (**c,d**) The generation of stable cell lines in H460 and H1975 cells in which SUN2 was overexpressed or silenced was confirmed through qRT-PCR (**c**) and western blotting (**d**). GAPDH was used as the internal control in (**c**) The bars correspond to the mean ± SEM. (**e,f**) The cell proliferation of the indicated stable cell lines *in vitro* was measured at different time points, as indicated by the CCK-8 assay. The bars correspond to the mean ± standard error, and the P value was calculated using Student’s t-test. *P < 0.05, **P < 0.01. (**g,h**) The colony formation of the indicated stable cell lines *in vitro* was measured for 14 days, as described in the Methods. The bars correspond to the mean ± standard error, and the P value was calculated using Student’s t-test. P < 0.0001. (**h)** is a representative image. The full-length blots are presented in Supplementary Fig. S9a and S9b.

**Figure 3:**
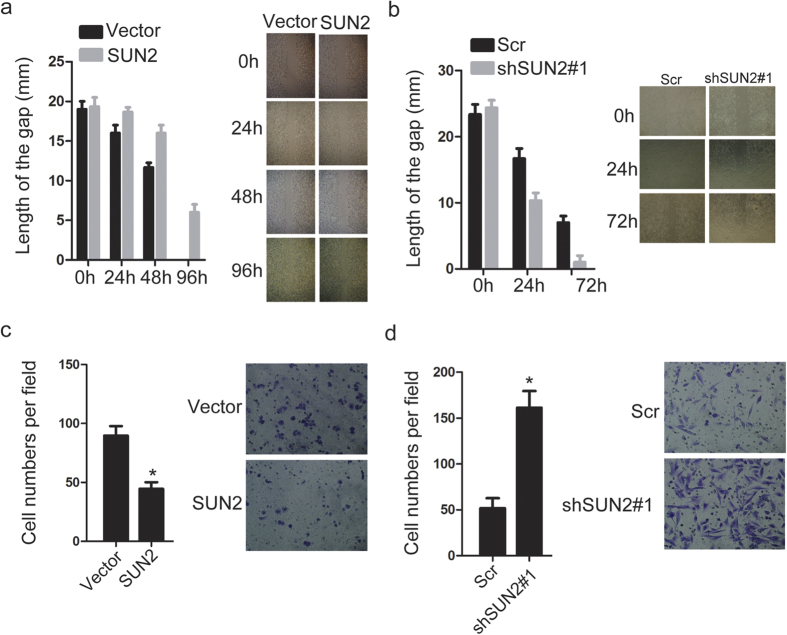
SUN2 suppresses the wound healing and migration of lung cancer cells. (**a,b**) Gap closure was dramatically suppressed or enhanced by overexpressing or silencing SUN2, respectively. The bars correspond to the mean ± SEM. (**c,d**) The migratory ability of the indicated stable cell lines was measured in the Transwell assay as described in the Methods. The bars correspond to the mean ± standard error, and the P value was calculated using Student’s t-test. *P < 0.05.

Given that SUN2 expression is down-regulated in lung cancer, we next asked whether it is involved in regulating the biological behaviors of lung cancer cells. First, we detected the expression of SUN2 in lung cancer cell lines using quantitative real-time PCR and western blotting (Fig. 2a,b). Based on the expression level of SUN2 in these lung cancer cells, we constructed stable cell lines with either ectopic expression of SUN2, in H460 cells, or silenced SUN2, in H1975 cells (Fig. 2c,d). We then investigated the effect of SUN2 on the biological behaviors of these cell lines. Cell proliferation and colony formation assays indicated that the ectopic expression of SUN2 significantly inhibited cell proliferation and colony formation abilities (Fig. 2e–h).In contrast, silencing SUN2 increased the cell proliferation ability of H1975 cells (Fig. 2f).To eliminate the possibility of off-target effects on shRNA#1, we selected another shRNA sequence for knockdown experiments. These results also showed that silencing SUN2 increased the proliferation of H1975 cells (Supplementary Fig. S4). We then examined whether SUN2 inhibited the migration of lung cancer cells. The ectopic expression of SUN2 suppressed H460 cell wound healing (Fig. 3a), whereas the knockdown of SUN2 promoted H1975 cell wound healing (Fig. 3b and Supplementary Fig. S5a).Moreover, using Transwell assays, we observed a notably reduced number of filtered H460 cells upon SUN2 overexpression (Fig. 3c) and an increased number of filtered H1975 cells after SUN2 silencing (Fig. 3d and Supplementary Fig. S5b). Taken together, these findings indicated that SUN2 inhibits lung cancer cell proliferation and migration.

### SUN2 induces apoptosis and enhances the chemotherapy sensitivity of lung cancer cells to cisplatin

**Figure 4:**
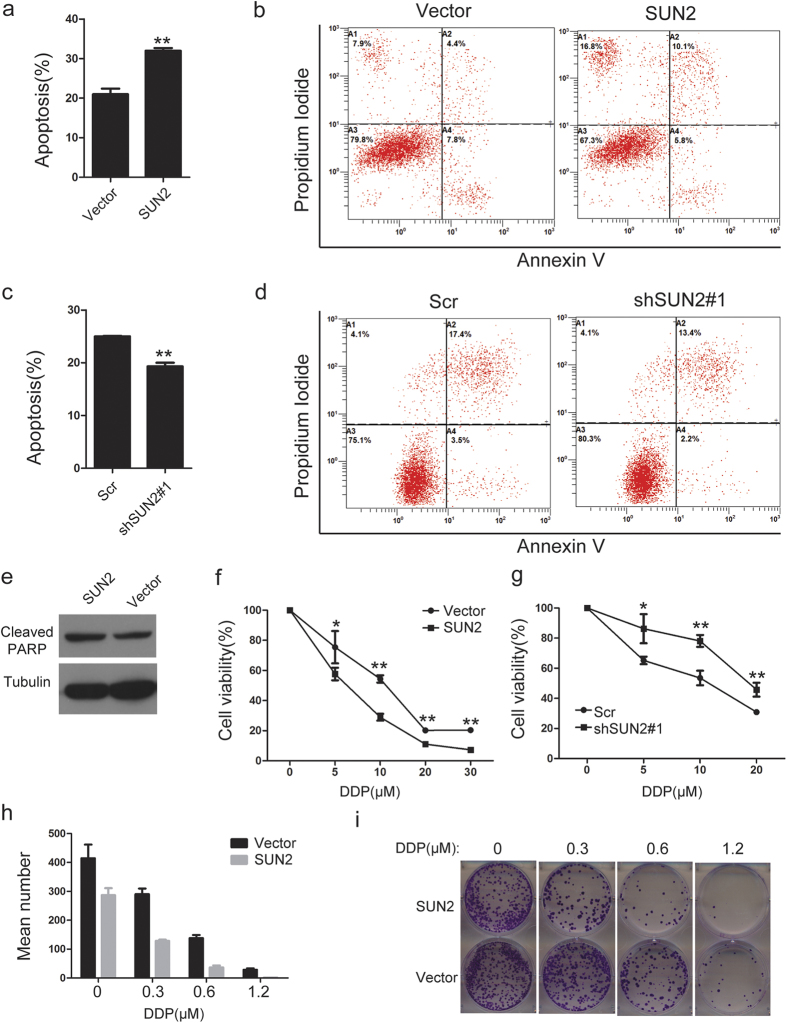
SUN2 promotes lung cancer cell apoptosis and increases chemotherapy sensitivity. (**a,b**) The stable cell lines overexpressing the empty vector or SUN2 were treated with 10 μM DDP for 24 h and then subjected to annexin V-FITC and PI staining. Cell apoptosis was evaluated through FACS. The bars correspond to the mean ± standard error (n = 3), and the P value was calculated using Student’s t-test. **P < 0.01. (**c,d**) The stable cell lines expressing scrambled or SUN2-silencing RNA were treated with 10 μM DDP for 24 h and then subjected to annexin V-FITC and PI staining. Cell apoptosis was evaluated through FCAS. The bars correspond to the mean ± standard error (n = 3), and the P value was calculated using Student’s t-test. **P < 0.01. (**e**) The stable cells expressing empty vector or SUN2 were treated with 10 μM DDP for 24 h and subjected to western blotting. (**f,g**) The stable cells in which SUN2 was overexpressed or knocked down were treated with the indicated concentrations of DDP for 24 h, and cell viability was assessed with the CCK-8 assay. The bars correspond to the mean ± standard error (n = 3), and the P value was calculated using Student’s t-test. *P < 0.05; **P < 0.01 (**h,i**) The stable cells expressing empty vector or SUN2 were evaluated using the colony formation assay after incubation with DDP (0.3, 0.6 and 1.2 μM) or vehicle (0 μM) (n = 3). The full-length blots are presented in Supplementary Fig. S9c.

Because SUN2 inhibits lung cancer cell proliferation, we next investigated whether SUN2 affects the apoptosis of lung cancer cells. The propidium iodide-annexin V assay was performed to evaluate the effect of SUN2 on cell apoptosis. As shown in Fig. 4a,b, the overexpression of SUN2 increased the apoptosis rate in lung cancer cells compared with the control cells. In contrast, the knockdown of SUN2 reduced lung cancer cell apoptosis (Fig. 4c,d and Supplementary Fig. S6a).Moreover, the overexpression of SUN2 increased the cleavage of PARP, a well-known marker of cell apoptosis (Fig. 4e).Because SUN2 induces lung cancer cell apoptosis, we sought to explore the association of SUN2 expression with cisplatin sensitivity *in vitro*. The stable cell lines (Fig. 2c,d and Supplementary Fig. S4a) were treated with increasing concentrations of cisplatin. The forced expression or deletion of SUN2 increased or decreased, respectively, the sensitivity of lung cancer cells to cisplatin, as shown by the cell viability assay(Fig. 4f,g and Supplementary Fig. S6b).The colony formation assay also indicated that overexpression of SUN2 decreased cell growth and increased the sensitivity of H460 cells to cisplatin (Fig. 4h,i).Taken together, these findings indicated that SUN2 induces lung cancer cell apoptosis and increases their chemotherapy sensitivity.

### SUN2 suppresses the Warburg effect in lung cancer cells

**Figure 5:**
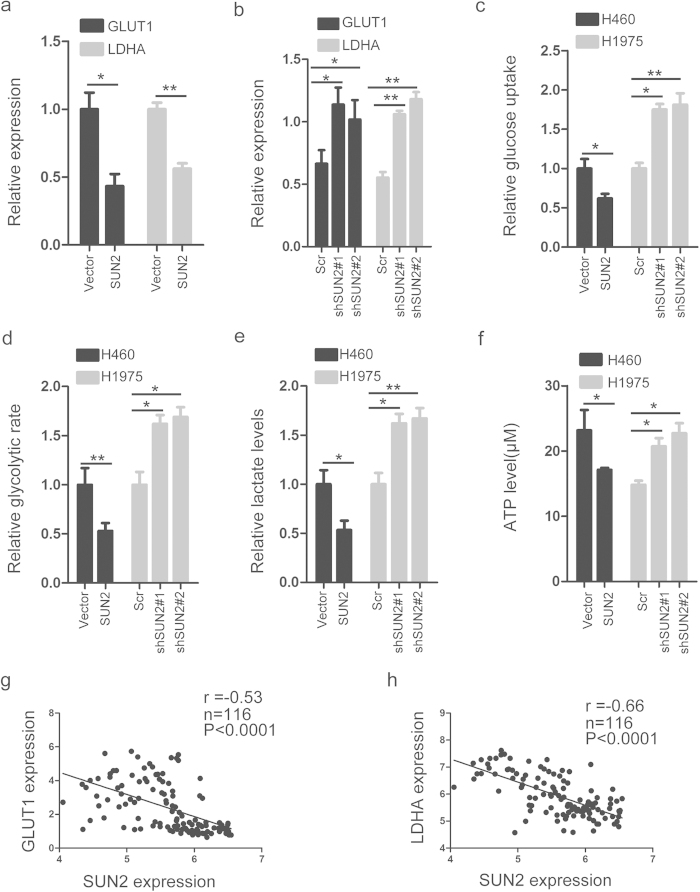
SUN2 inhibits the Warburg effect in lung cancer. (**a,b**) The mRNA levels of GLUT1 and LDHA were determined via qRT-PCR in the stable cell lines in which SUN2 was expressed or knocked down. GAPDH was used as an internal control. The data are presented as the mean ± standard error (n = 3). *P < 0.05; **P < 0.01 (Student’s t test). (**c–f**) Glucose uptake levels, the glycolytic rate, lactate production and ATP concentrations were measured in the stable cell lines in which SUN2 was expressed or knocked down, as described in the Methods. The data are presented as the mean ± standard error (n = 3). *P < 0.05; **P < 0.01(Student’s t test). (**g**) A significant negative correlation between SUN2 expression and GLUT1 expression was observed in the tissues. The raw data were obtained from the Oncomine database. (**h**) A significant negative correlation between SUN2 expression and LDHA expression was observed in the tissues. The raw data were obtained from the Oncomine database.

The majority of cancer cells preferentially use aerobic glycolysis instead of oxidative phosphorylation to meet their increased energetic and biosynthetic demands, a phenomenon known as the Warburg effect^17^. The Warburg effect is associated with tumor development and can fulfill the energetic demands of the membrane transport activities required for proliferation and migration^26^. GLUT1 and LDHA, the two key genes associated with the Warburg effect, are required for glucose uptake and the conversion of pyruvate to lactate, respectively^27^. The expression of GLUT1 and LDHA is higher in lung cancer cells than paired normal lung tissues according to the Oncomine database^20^ (Supplementry Fig. S7). To address the issue of whether SUN2 regulates the Warburg effect, we investigated the effect of SUN2 on GLUT1 and LDHA expression in lung cancer cells (Fig. 2c,d and Supplementary Fig.S4a). As predicted, overexpression or knockdown of SUN2 decreased or increased GLUT1 and LDHA expression (Fig. 5a,b), respectively. Furthermore, the overexpression of SUN2 significantly inhibited the Warburg effect in H460 cells, whereas knockdown of SUN2 significantly promoted the Warburg effect in H1975 cells(Fig. 5c–f).In addition, we observed significant inverse correlations between SUN2 and GLUT1 and between SUN2 and LDAH in the tissues (Fig. 5g,h). These findings indicate that SUN2 suppresses the Warburg effect by repressing the expression of GLUT1 and LDAH in lung cancer cells.

### SIRT5 is a negative regulator of SUN2 in lung cancer

**Figure 6:**
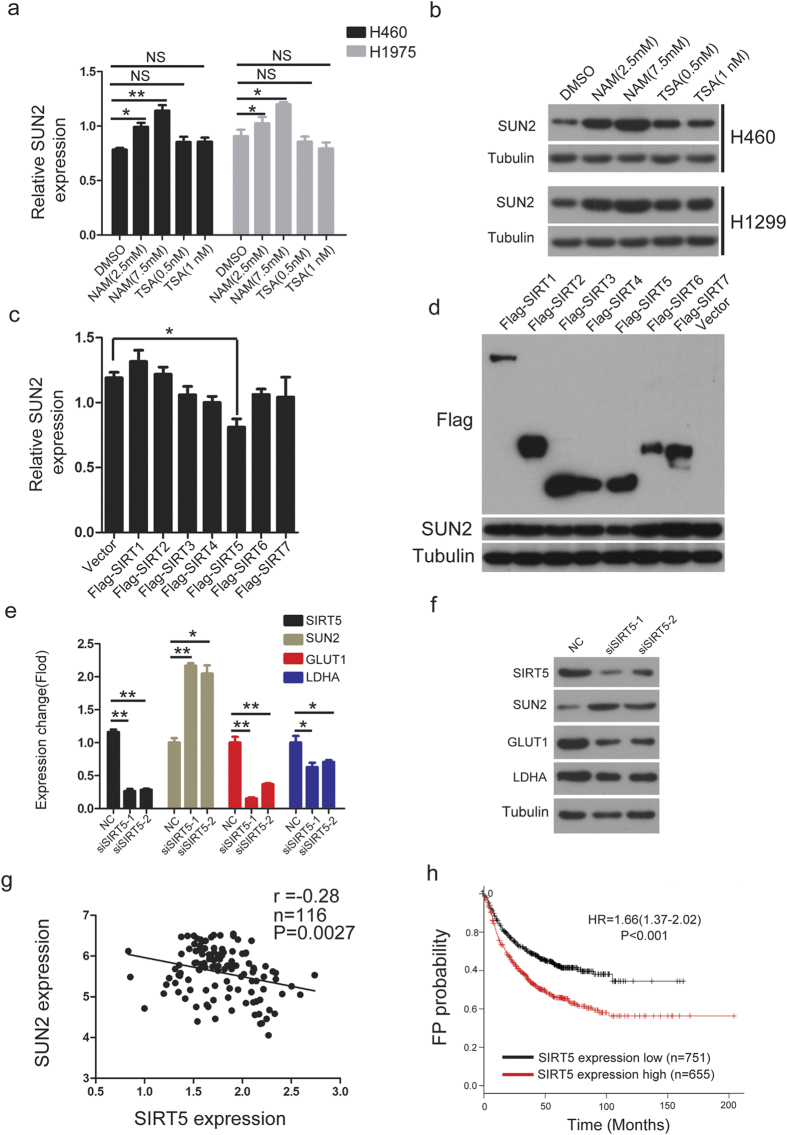
SIRT5 is a negative regulator of SUN2 in lung cancer. (**a,b**) The mRNA and protein levels of SUN2 were determined through qRT-PCR and western blotting, respectively, in H460 and H1299 cells treated with trichostatin A or nicotinamide for 24 h. GAPDH was used as an internal control in (**a**) The bars correspond to the mean ± standard error (n = 3), and the P value was calculated using Student’s t-test. *P < 0.05; **P < 0.01 (**c,d**) H1299 cells were transfected with empty vector or Flag-tagged SIRTs, as indicated, for 48 h. The mRNA levels of SUN2 were evaluated through qRT-PCR (**c**), and the protein levels of SUN2 and the SIRTs were determined via western blotting (**d**). The bars correspond to the mean ± standard error (n = 3), and the P value was calculated using Student’s t-test. *P < 0.05. (**e,f**) H1299 cells were transfected with a negative control (NC) or two effective siRNAs targeting SIRT5 for 48 h. The cells were then harvested to determine the mRNA and protein levels of SIRT5, SUN2, GLUT1 and LDHA via qRT-PCR (**e**) and western blotting (**f**), respectively. GAPDH was used as an internal control in **e**. The bars correspond to the mean ± standard error (n = 3), and the P value was calculated using Student’s t-test. *P < 0.05; **P < 0.01. (**g**) A significant negative correlation between SIRT5 expression and SUN2 expression was shown in the tissues. The raw data were obtained from the Oncomine database. (**h**) Lung cancer patients with high SIRT5 expression exhibited significantly shorter first progression-free survival (FP) than those with low expression, P < 0.001. The full-length blots are presented in Supplementary Fig. S9d to S9f.

To investigate the mechanism underlying the reduction of SUN2 levels in lung cancer cells, we analyzed the promoter sequence of SUN2 and found a CpG island in this region. Then, we performed bisulfate genomic sequencing to determine the methylation status of the CpG island in five lung cancer cell lines, including H1975, HCC827, H460, H1650 and H1299 (Supplementary Fig. S8a). However, methylation of the CpG island was nearly undetectable in these cell lines. Additionally, restoration of SUN2 mRNA was not observed in lung cancer cells after treatment with 5-Aza-dC, a DNA methylation inhibitor (Supplementary Fig. S8b). We next treated H460 and H1299 cells with the histone deacetylase (HDAC) inhibitors trichostatin A (TSA) and nicotinamide (NAM) for 24 h; these reagentsare inhibitors of the class I and II HDACs and class III HDACs^28^,^29^, respectively. We found that the addition of nicotinamide, but not TSA, increased the mRNA and protein levels of SUN2, indicating that SUN2 may be regulated by the sirtuin family (Fig. 6a,b). We then sought to determine which sirtuin member was responsible for the repression of SUN2 expression through the ectopic expression of SIRTs. As shown in Fig. 6c,d, only ectopic SIRT5 significantly suppressed SUN2 expression. In contrast, knockdown of SIRT5 using two effective siRNAs significantly increased SUN2 expression (Fig. 6e,f). In addition, the knockdown of SIRT5 decreased GLUT1 and LDAH expression (Fig. 6e,f), which suggested that SIRT5 promoted the Warburg effect by repressing SUN2 expression. We also found that SIRT5 expression was inversely correlated with SUN2 at the mRNA level in tissues (Fig. 6g). Moreover, patients with high SIRT5 expression exhibited poor FP survival (Fig. 6h). These results indicated that SIRT5 acts as a negative regulator of SUN2 in lung cancer.

## Discussion

A better understanding of the mechanisms underlying lung cancer development, progression and therapy resistance is urgently needed to design novel effective therapies for this deadly cancer. SUN2 has been shown to act as a tumor suppressor in central nervous system embryonal tumors and breast cancer^14^,^15^. In this report, we demonstrated for the first time that SUN2 is a key player in lung cancer progression by inhibiting the Warburg effect. We also reported that SIRT5 is at least partly responsible for the down-regulation of SUN2 in lung cancer cells. This novel SIRT5/SUN2 axis may be useful for the development of new strategies for treating patients with lung cancer.

We demonstrated that the mRNA and protein levels of SUN2 were significantly decreased in lung cancer by performing data mining and immunohistochemistry analysis. Low *SUN2* expression was shown to be associated with poor outcomes in patients with lung cancer, indicating that the loss of SUN2 is likely to present prognostic value. To explore the mechanism underlying the decrease in SUN2 expression in lung cancer cells, we first examined the methylation status of the SUN2 promoter, given that hypermethylation of the promoters of many tumor suppressor genes leads to a decrease in their expression in cancer^30^. However, despite the presence of a CpG island in the SUN2 promoter region, methylation of this CpG island was almost undetectable in the cell lines. Finally, we provided evidence showing that SIRT5 is at least one of the negative regulators of SUN2. SIRT5, a mitochondrial sirtuin, is present both inside the mitochondria and within the cytosol and nucleus.SIRT5 has been reported to facilitate cancer cell growth and drug resistance in non-small cell lung cancer^31^. Our results suggested that SIRT5 may play an oncogenic role by negatively regulating SUN2.

Our results demonstrated that SUN2 suppresses cell proliferation and migration, promotes apoptosis and enhances chemotherapy sensitivity to cisplatin in lung cancer. The Warburg effect is one of the hallmarks of cancer cells, including lung cancer cells^32^. The switch from oxidative phosphorylation to glycolysis provides a critical mechanism for cancer cells to meet their dramatically increased energetic and biosynthetic demands to support their rapid growth and proliferation^17^. GULT1 and LDHA are important regulators of the Warburg effect, and both are involved in early carcinogenesis and tumor progression^33^. In this report, we showed that SUN2 decreases glucose uptake, the glycolytic rate and lactate production in lung cancer cells by decreasing the expression of GLUT1 and LDHA. This regulatory effect is confirmed by the observation that SUN2 expression shows a significant negative correlation with GLUT1 and LDHA expression in tissues. These results suggest that SUN2 may exert its tumor suppressor function by suppressing the Warburg effect.

There are significant differences in the nuclear architecture between cancer and normal cells. The LINC complex, which maintains nuclear structure by connecting the nuclear lamina to the cytoskeleton, exhibits diverse functions, playing roles in determining the nuclear structure, nuclear positioning, cell migration, the cell cycle, cell proliferation and genome integrity^15^. Loss of the LINC complex, which reduces nuclear and cellular rigidity and consequently increases the tissue fluidity that is crucial for invasive activity, is associated with human diseases including cancer^10^,^15^. For example, nesprins, which are components of the LINC complex, are involved in critical cellular processes that, in the case of malfunction, contribute to the formation of cancer and might represent novel targets for cancer diagnosis or therapeutic interventions^10^,^34^. Additionally, the LINC complex decreases the adhesion of cancer cell invadopodia to the matrix^35^. Revach OY *et al*. showed that knockdown of the LINC complex components nesprin 2 and SUN1 leads to a substantial increase in the prominence of the adhesion domains at the opposite end of the invadopodia^35^. In addition, loss of the LINC complex may regulate cell proliferation and promote cancer progression; consistent with this association, nesprin-1 and nesprin-2 play roles in cell proliferation^15^,^36^. Our results revealed that SUN2, another component of the LINC complex, plays an important role in inhibiting cell proliferation and migration in lung cancer.

In summary, our findings demonstrate that SUN2 plays a tumor suppressor role by repressing the Warburg effect and could serve as a clinical predictor in lung cancer. We also highlight that SUN2 expression derangements are involved in the SIRT5-mediated machinery, all of which offer potential avenues for the treatment of this fatal disease. Together, these data provide new insights for understanding the molecular basis underlying this deadly malignancy.

## Methods

### Cells and Reagents

Seven human lung cancer cell lines (H358, H460, HCC827, H1650, H1299, H1975 and A549) were purchased from the American Type Culture Collection (ATCC) and cultured according to their instructions. All of the cell lines used in this study were authenticated through short-tandem repeat profiling less than 6 months ago, when this project was initiated, and the cells have not been in culture for more than 2 months. 5-AZA, trichostatin A and nicotinamide were purchased from Sigma-Aldrich.

### Plasmids

The full-length cDNA of human SUN2 was cloned into the pSin-puro vector. Flag-SIRT1, Flag-SIRT2, Flag-SIRT3, Flag-SIRT4, Flag-SIRT5, Flag-SIRT6 and Flag-SIRT7 were constructed by inserting a Flag tag into the SIRTs N-terminus in a pcDNA3.1 (+) vector. All recombinant plasmids were verified through DNA sequencing.

### Antibodies

Human anti-cleaved PARP and anti-flag antibodies were obtained from Cell Signaling Technology. Anti-tubulin was procured from Bioworld Company. Anti-GLUT1 and anti-LDHA antibodies were purchased from Santa Cruz Biotechnology. Anti-SUN2 (HPA001209) and anti-Sirt5 (HPA021798) antibodies were obtained from Sigma-Aldrich.

### RNA interference and stable lines

Effective siRNA oligonucleotides targeting SIRT5#1 and #2 with sequences of 5′-UGUCCAGCUUUAUCAGGAA-3′ and 5′-GGAGAUCCAUG GUAGCUUA-3′, respectively, were synthesized by RuiBo (Guangzhou). Approximately 2 × 10^5^ H1299 cells per well were seeded in a 60-mm culture dish on the day before transfection. Transfection with 50 nmol of siRNA was performed according to the manufacturer’s instructions using the Lipofectamine^TM^ RNAiMAX transfection reagent (Life Technologies). After transfection for 48 h, qRT-PCR and western blot assays were performed. Cell lines stably expressing scrambled or SUN2 short hairpin RNA (shRNA) were established using the Sigma-Aldrich shRNA system according to the manufacturer’s instructions. The oligonucleotides for human SUN2 shRNA#1 and #2 were 5′- GCAAGACTCAGAAGACCTCTT -3′ and 5′-GCCTATTCAGACGTTTCACTT-3′, respectively. pSin-puro delivering SUN2 or empty vector was co-transfected with pMD.2G and psPAX2 into HEK-293T cells for 48 h. The recombinant viruses were subsequently collected and added to H460 cells cultured with 8 μg/ml polybrene for 24 h. The stable lines were selected with 1 μg/ml puromycin for two weeks.

### RNA extraction and qRT-PCR

These procedures were performed as previously described^37^,^38^. Briefly, total RNA was isolated using the TRIzol reagent (Invitrogen) according to the manufacturer’s instructions. First-strand cDNA was synthesized using the Revert Aid^TM^ First Strand cDNA Synthesis Kit (MBI Fermentas). The primers employed for amplifying SUN2, SIRT5, GLUT1, LDHA and GAPDH were validated. SUN2 primers are as follows: F 5′-CCAGTCAC CCCGAGTCATC-3′ and R 5′-ATGCTCTAAGGTAACGGCTGT-3′. GLUT1 primers are as follows: F 5′-GGCCAAGAGTGT GCTAAAGAA-3′ and R 5′-ACAGCGTTGATGCCAGACAG-3′. LDHA primers are as follows: F 5′-TTGACCTACGTGGCTTGGAAG-3′ and R 5′-GGTAACGGAATCGGGCTGA AT-3′. SIRT5 primers are as follows: F 5′-GCCATAGCCGAGTGTGAGAC-3′ and R 5′-CAACTCC ACAAGAGGTACATCG-3′. GAPDH primers are as follows: F 5′-ACAGTCAGCCGCATCTTCTT-3′ and R 5-GACAAGCTTCCCGTTCTCAG-3′.

### Cell proliferation and cell viability assays

*In vitro* cell proliferation and cell viability were assessed using the CCK-8 assay. For cell proliferation, cells were seeded in 96-well plates at a density of 1,000 cells/well and incubated for 1, 2, 3, 4, or 5 days; For cell viability, cells were seeded in 96-well plates at a density of 5,000 cells/well and treated with DDP for 24 hours. Ten microliters of the CCK-8 reagent (Cell Counting Kit-8, Beyotime, China) was then added to each well, followed by incubation for 1.5 h. The absorbance value (OD) of each well was measured at 450 nm. For each experimental condition, 6 wells were used.

### Transwell assays

For the Transwell migration assay, 1.0 × 10^5^ (H460) or 7 × 10^4^ (H1975) cells in 200 μl of serum-free RPMI 1640 were added to cell culture inserts with an 8-μm microporous filter, without an extracellular matrix coating (Becton Dickinson Labware, Bedford,MA). RPMI 1640 medium containing 10% FBS was then added to the bottom chamber. After 24 h of incubation, the cells on the lower surface of the filter were fixed, stained, and examined using a microscope. The number of migrated cells in three random optical fields (×100 magnification) from triplicate filters was averaged.

### DNA methylation analysis

Genomic DNA was extracted from lung cancer cell lines using the QIAamp DNA Mini Kit (Qiagen, Hilden, Germany). A total of 1μg of DNA was treated with sodium bisulfite using the Zymo DNA Modification Kit (Zymo Research, Hornby, Canada). Bisulfite genomic sequencing was performed to assess the methylation levels of nineteen CpG sites spanning from −290 to −27 of variant 1 within the SUN2 promoter. The nucleotide sequences of the primers used in bisulfate genomic sequencing were as follows: F 5′-TTAGAGTTGTTTTAGGTGTTTTGAG-3′ and R 5′-AACCCATTAATCAAACCCC-3′.

For the demethylation treatment, the lung cancer cells were seeded at a density of 1 × 10^5^ cells/ml in 100-mm dishes for 24 h, and the cells were treated with 10 μM 5-Aza, a DNA demethylating agent, every day for 5 days. Then, the cells were harvested and analyzed through real-time qRT-PCR.

### Western blotting

These procedures were performed as described previously^37^,^38^. The gels were run under the same experimental conditions and the original whole gel blots were included in the supplementary data.

### IHC and histological evaluation

This procedure was described previously^39^,^40^. The immunohistochemical analysis was performed on 3-μm sections. Primary antibody against SUN2 was diluted 1:2000 and was incubated with the samples at 4 °C overnight in a humidified container. After washing with PBS three times, the tissue slides were treated with a non-biotin horseradish peroxidase detection system according to the manufacturer’s instructions (Dako). The resultant IHC staining was evaluated by two independent pathologists majoring in lung cancer. The protein expression of SUN2 was evaluated using the semiquantitative IRS (immunoreactive score) scale according to Remmele and Stegner^41^.

### Colony formation assay

Cells were plated in 6-well culture plates at 250 cells per well. Each group included 3 wells. After incubation for 15 days at 37 °C, the cells were washed twice with PBS and stained with Giemsa solution. The number of colonies containing ≥50 cells was counted under a microscope.

### Wound-healing assay

Cell motility was assessed by measuring the movement of cells into a scraped cellular area created by a 200-μl pipette tube, and the spread of wound closure was observed after 24, 48 and 96 h for H460 cells (24h and 72 h for H1975 cells). The cells were photographed under a microscope.

### Flow cytometry

Apoptosis analysis was conducted with an Annexin V-FITC Apoptosis Detection Kit (KeyGen Biotech, China) according to the manufacturer’s protocol. The percentage of apoptotic cells was determined using FACS flow cytometer equipped software (BECKMAN).

### Analysis of glucose uptake, the glycolytic rate and lactate production in cells

Glucose uptake levels were determined by measuring the uptake of ^3^H-2-deoxyglucose. Briefly, cells cultured in 12-well plates were pre-incubated in glucose-free media for 30 min before adding ^3^H-2-deoxyglucose (1 μCi/well) to the cells. After incubation for 30 min, the cells were washed with PBS and lysed in 1% SDS. The radioactivity of the cell lysates was determined in a liquid scintillation counter and normalized to the protein concentrations of the cell lysates. Cellular glycolytic rates were measured by monitoring the conversion of 5-^3^H-glucose to ^3^H_2_O. Briefly, the cells (1 × 10^6^) were collected and washed once in PBS before they were resuspended in 1 mL of Krebs buffer without glucose for 30 min at 37 °C. The cells were collected and resuspended in 0.5 mL of Krebs buffer containing 10 mM glucose and 5 μCi of 5-^3^H glucose for 1 h at 37 °C. Triplicate 100 μL aliquots were transferred to uncapped PCR tubes containing 100 μL of 0.2 N HCL, and the tubes were transferred to scintillation vials containing 0.5 mL of H_2_O. The scintillation vials were then sealed and left for 48 h. The amounts of diffused and undiffused ^3^H were subsequently determined in a liquid scintillation counter. Lactate production levels were measured using a Lactate Assay Kit (BioVision, Milpitas, CA, USA). Cells were plated in 100-mm culture dishes at a density of 1 × 10^6^ cells/plate. After incubation for 24 h, the culture medium was replaced with FBS-free DMEM. The lactate levels in the culture medium were examined with a Lactate Assay Kit and normalized to the number of cells.

### ATP measurements

ATP measurements were obtained using an ATP determination kit (Beyotime Biotechnology, China).The ATP content was determined based on comparison with a concurrent standard curve.

### Clinical Data Set Analysis

The correlations of SUN2 or SIRT5 with the clinical characteristics or survival of lung cancer patients’ were analyzed using the online KMplot database (http://www.KMplot.com).

### Study approval

The use of human lung cancer tissues was reviewed and approved by the ethical committee of Jiangxi cancer Hospital and was performed in accordance with the approved guidelines. The informed consent has been obtained.

### Statistical analysis

All statistical analyses were performed using SPSS for Windows, version 16.0 (SPSS). Pearson’s correlation analysis was performed to assess the relationships between SIRT5, SUN2, GLUT1 and LDHA in the tissues using mRNA expression data from Oncomine. All values from the *in vitro* assays are expressed as the mean ± SD or SEM of at least three independent experiments or replicates. P values were calculated using the two-tailed Student’s test. A p value < 0.05 is considered statistically significant.

## Additional Information

**How to cite this article**: Lv, X.-b. *et al*. SUN2 exerts tumor suppressor functions by suppressing the Warburg effect in lung cancer. *Sci. Rep.* **5**, 17940; doi: 10.1038/srep17940 (2015).

## Supplementary Material

Supplementary Information
