# Supplementary material for: SUN2 exerts tumor suppressor functions by suppressing the Warburg effect in lung cancer
Source: Sci Rep. 2015 Dec 10;5:17940. doi: 10.1038/srep17940 (PMC4674702; doi:10.1038/srep17940)
Supplement: Supplementary Information [file srep17940-s1.doc]

**SUN2 exerts tumor suppressor functions by suppressing the Warburg effect in lung cancer**

Xiao-bin Lv^1#^,Lijuan Liu^2#^,Chun Cheng^1#^,Bentong Yu^3^,Longxin Xiong^1^, Kaishun Hu^4^, Jianjun Tang^2^, Lei Zeng^2^,Yi Sang^1*^

^1^Nanchang Key Laboratory of Cancer Pathogenesis and Translational Research, Center Laboratory, the Third Affiliated Hospital, Nanchang University, Nanchang, China.

^2^Department of Pharmacy, Jiangxi cancer Hospital, Nanchang, China.

^3^Department of cardiothoracic surgery, the First Affiliated Hospital, Nanchang University, Nanchang, China.

^4^Guangdong Provincial Key Laboratory of Malignant Tumor Epigenetics and Gene Regulation, Medical Research Center, Sun Yat-Sen Memorial Hospital, Sun Yat-Sen University, Guangzhou, China.

^*^**Correspondence to:** Yi Sang, Nanchang Key Laboratory of Cancer Pathogenesis and Translational Research, Center Laboratory, the Third Affiliated Hospital of Nanchang University, No.128, Xiangshan North Road, Nanchang, China. E-mail: [sangyi10@foxmail.com](mailto:sangyi10@foxmail.com)

#These authors contributed equally to this work.

**
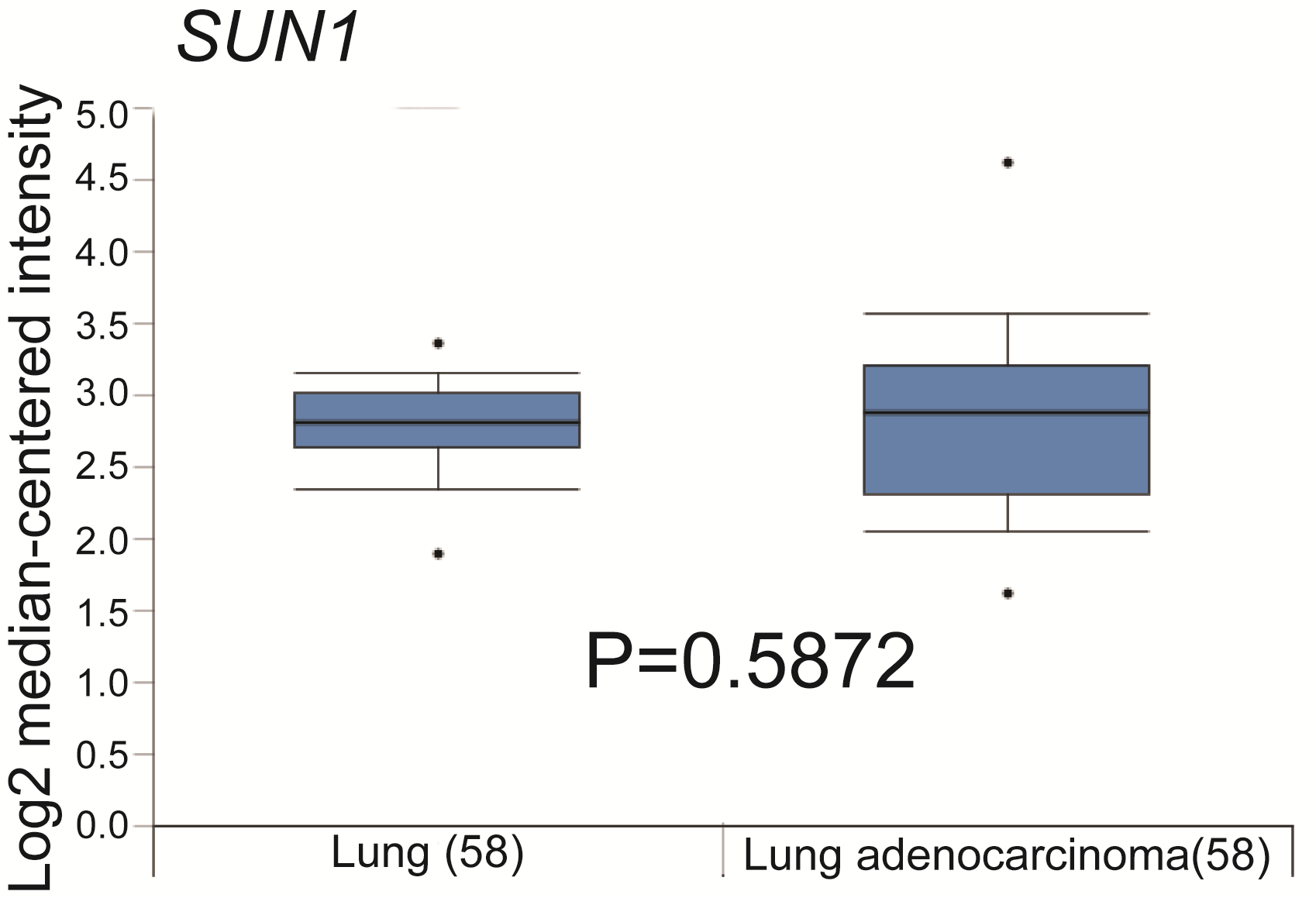
**

**Supplementary Figure 1. *SUN1* expression decreases in lung cancer.** Meta-analysis of *SUN1* expression in lung cancer performed using the Oncomine database. Box plots showing the lack of significantly different *SUN1* expression between normal lung tissues and lung cancer tissues. The P value was calculated according to the raw data using Student’s t-test.


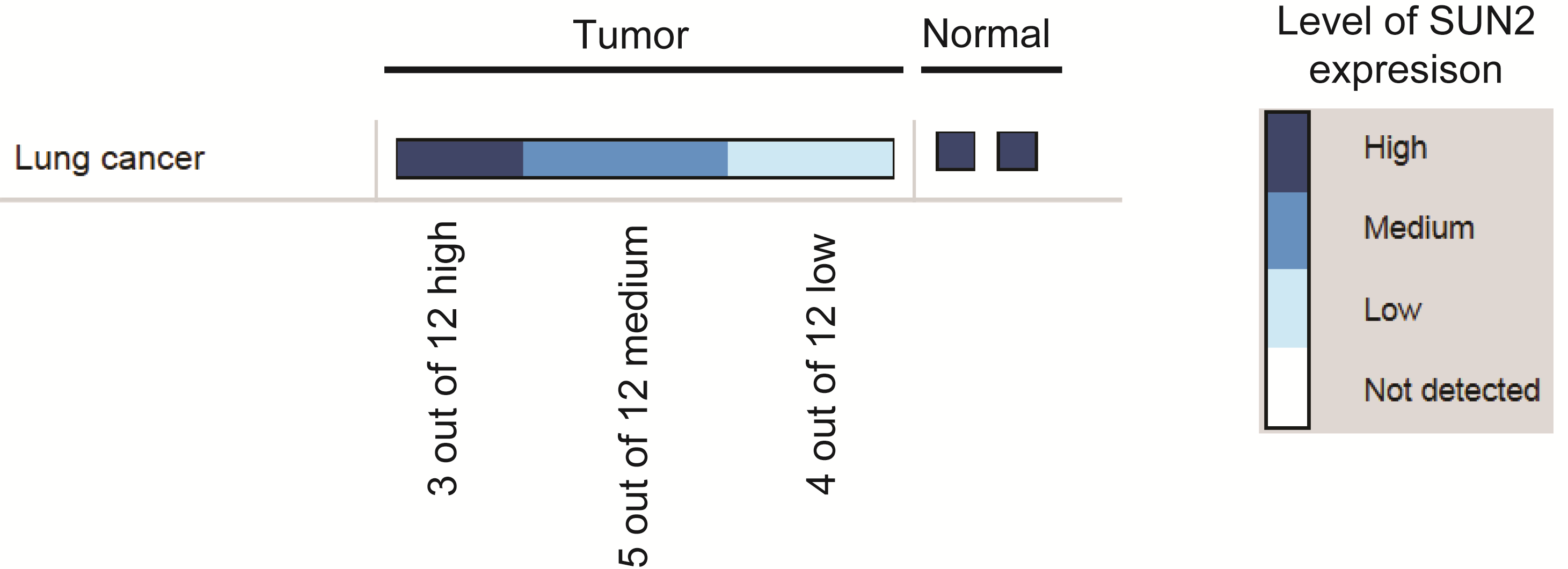


**Supplementary Figure 2. SUN2 expression decreases in lung cancer.** Analysis of SUN2 protein expression in lung cancer performed using the Protein Atlas database (<http://www.proteinatlas.org/ENSG00000100 242-SUN2/cancer>)


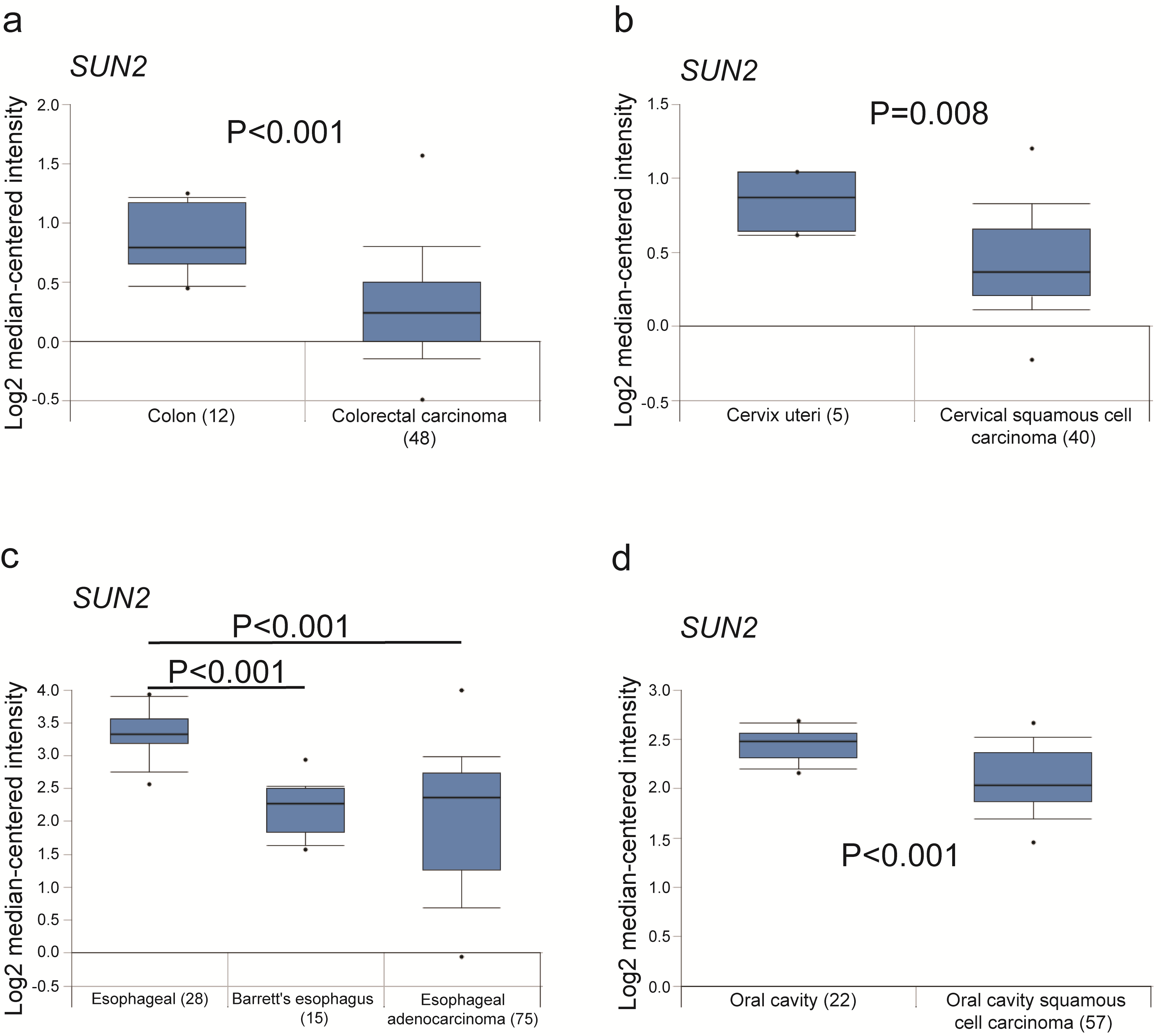


**Supplementary Figure 3. *SUN2* expression is decreased in various cancers.** (**a-d**) Meta-analysis of *SUN2* expression in various cancers performed using the Oncomine database. Box plots showing decreased expression of *SUN2* in cancer tissues. The P value was calculated according to the raw data using Student’s t-test.


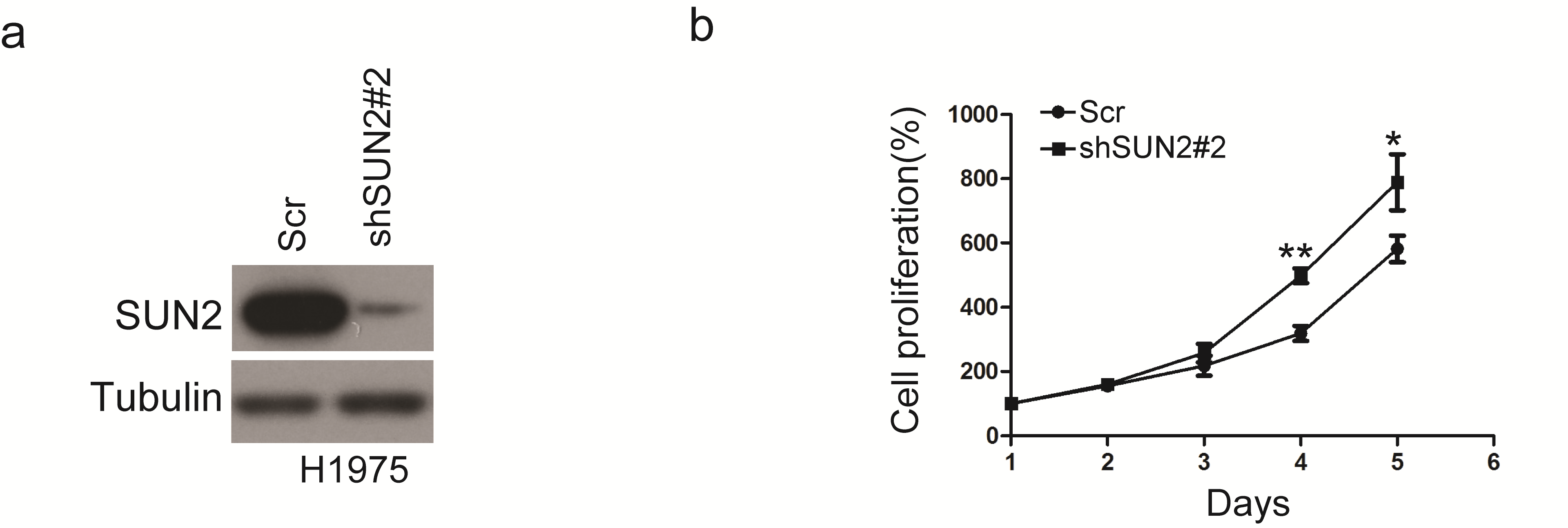


**Supplementary Figure 4. Knockdown of SUN2 using the shRNA#2 sequence promotes lung cancer cell proliferation.** (**a**)The knockdown of SUN2 using the shRNA#2 sequence was confirmed through western blotting. (**b**)The cell proliferation of the indicated stable cell lines *in vitro* was measured at different time points, as indicated by the CCK-8 assay. The bars correspond to the mean± standard error, and the P value was calculated using Student’s t-test. *P<0.05, **P<0.01.


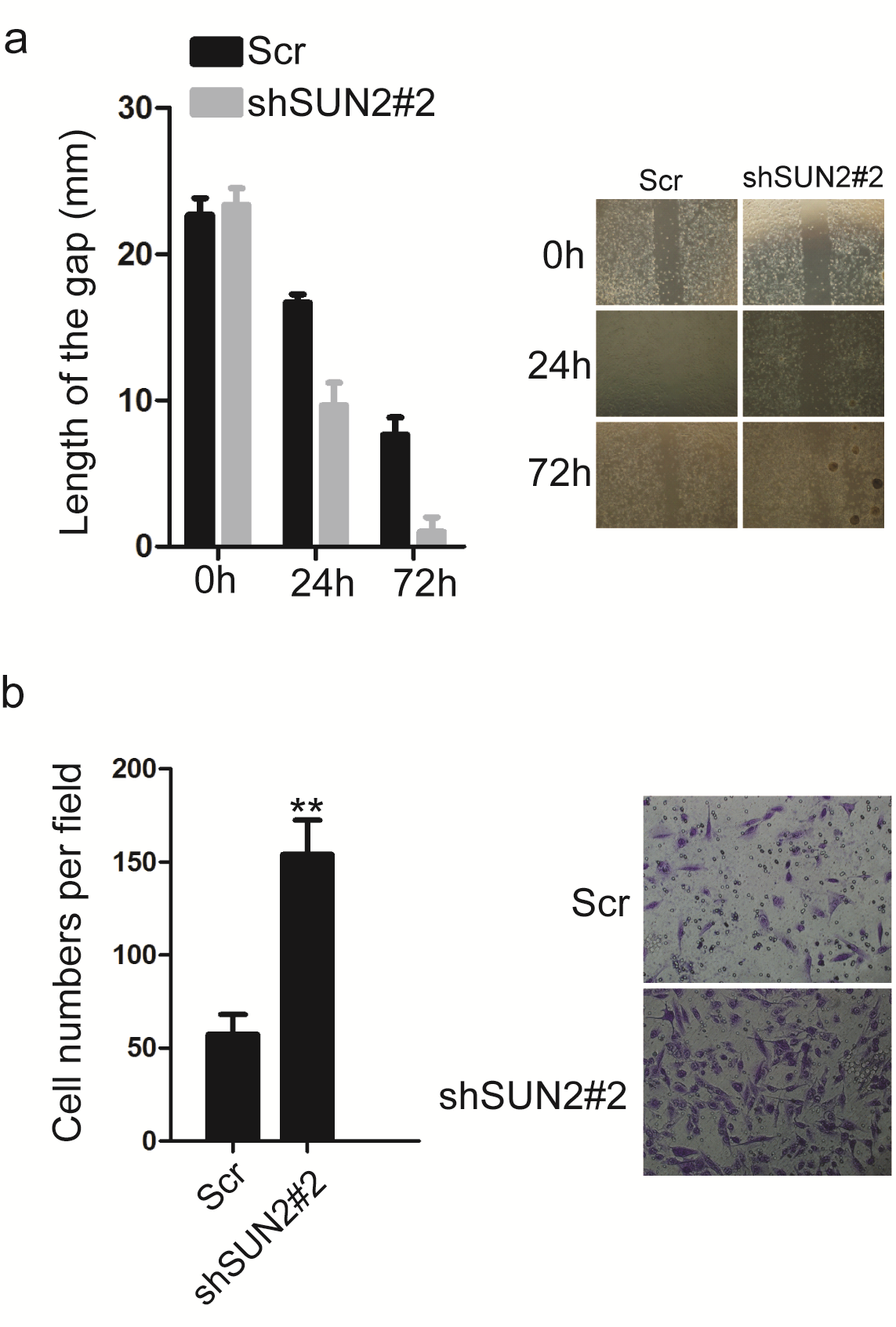


**Supplementary Figure 5. Knockdown of SUN2 using the shRNA#2 sequence promotes the wound healing and migration of lung cancer cells.** (**a**) Gap closure was dramatically enhanced by knocking down SUN2. The bars correspond to the mean ± SEM. (**b**) The migratory ability of the indicated stable cell lines was measured via the Transwell assay, as described in the Methods. The bars correspond to the mean± standard error, and the P value was calculated using Student’s t-test. **P<0.01.


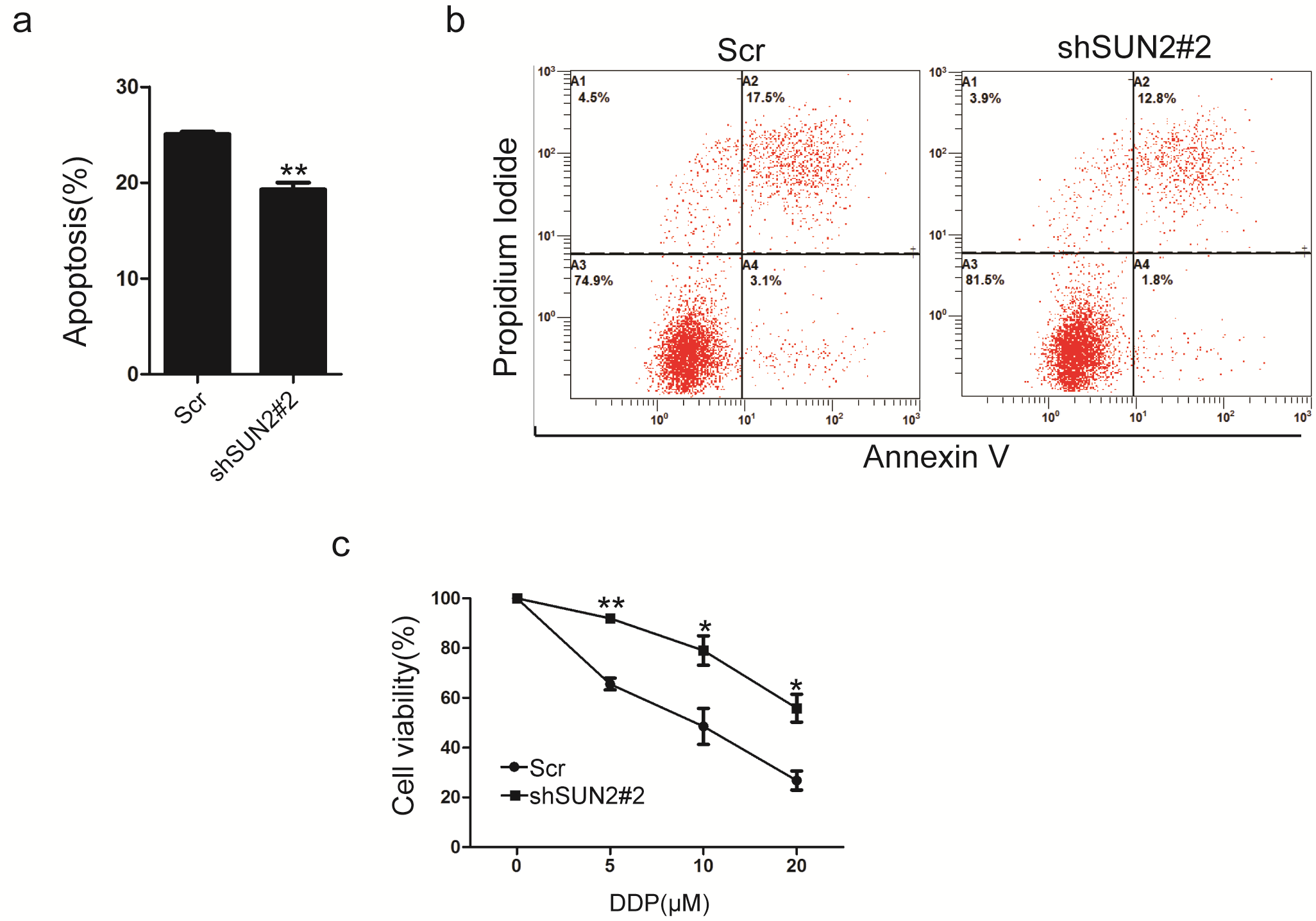


**Supplementary Figure 6. Knockdown of SUN2 using the shRNA#2 sequence inhibited apoptosis and decreased chemotherapy sensitivity.** (**a**)The stable cell lines expressing scrambled or SUN2 silencing RNA were treated with 10 μM DDP for 24 h and then subjected to annexin V-FITC and PI staining. Cell apoptosis was evaluated through FACS. The bars correspond to the mean± standard error (n = 3), and the P value was calculated using Student’s t-test. **P<0.01. (**b**) The stable cells in which SUN2 was knocked down were treated with the indicated concentrations of DDP for 24 h, and cell viability was assessed via the CCK-8 assay. The bars correspond to the mean± standard error, and the P value was calculated using Student’s t-test. *P<0.05, **P<0.01.


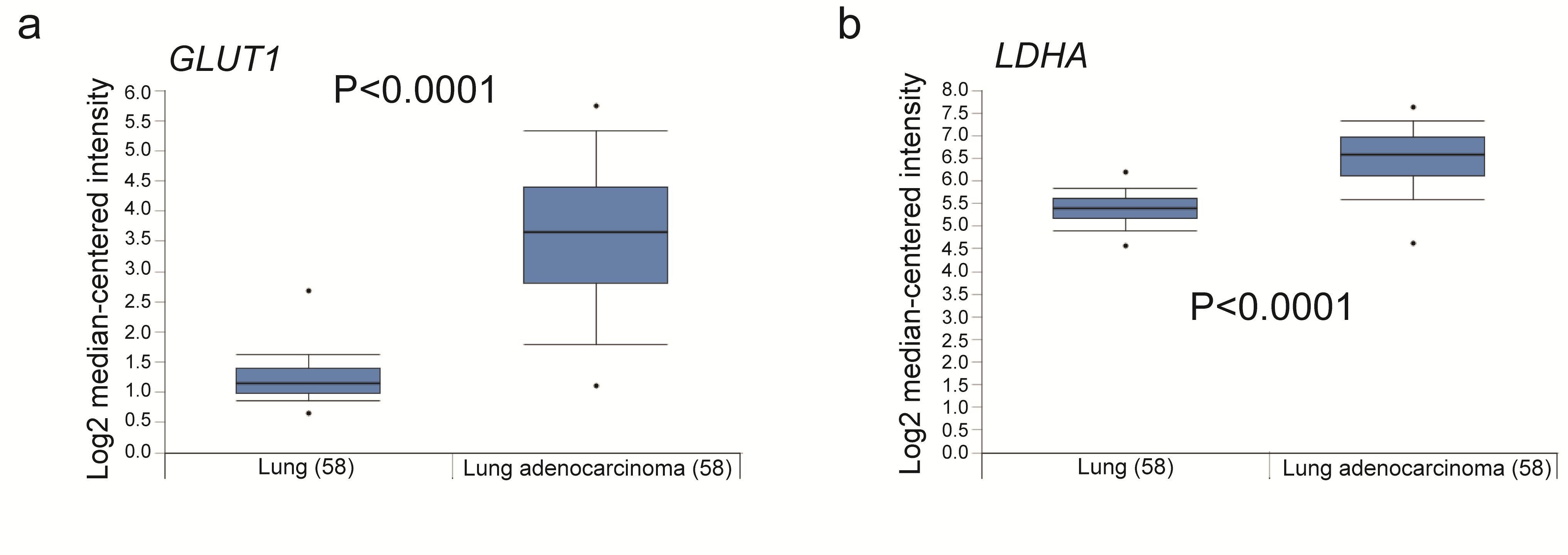


**Supplementary Figure 7. GLUT1 and LDHA expression is up-regulated in lung cancers.** **(a, b**) Meta-analysis of GLUT1 (**a**) and LDHA (**b**) expression performed using Oncomine (http://www.oncomine.org). Box plots showing increased expression of GLUT1 or LDHA during tumorigenesis in lung adenocarcinoma datasets. The y axis represents the log2 median-centered intensity (normalized expression). The shaded boxes represent the interquartile range (25^th^–75^th^ percentile). The whiskers represent the 10^th^–90^th^ percentile. The bars denote the median. The P value was calculated according to the raw data using Student’s t-test.


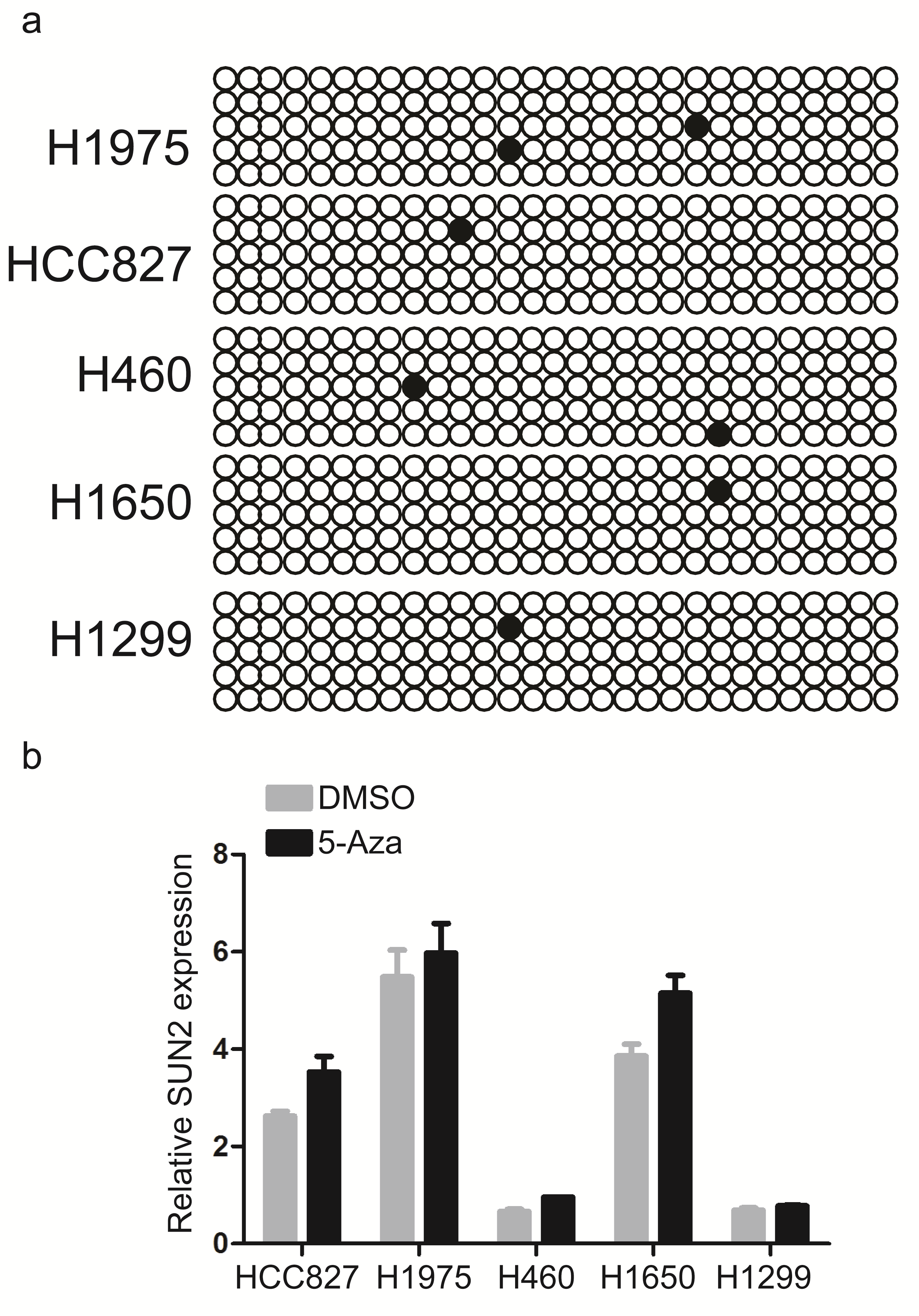


**Supplementary Figure 8. The methylation of the CpG sites in the SUN2 promoter is nearly undetectable in lung cancer cells.** (**a**) The SUN2 promoter was amplified from bisulfite-converted DNA and cloned into the pMD18T TA-cloning vector. The status of the methylated cytosine residues within the SUN2 promoter was evaluated by sequencing 5 colonies randomly picked from the indicated lung cancer cell lines. Open ovals indicate unmethylated CpG sites, and solid ovals indicate methylated sites. (**b**) The relative mRNA levels of SUN2 normalized to the levels of GAPDH in the indicated cell lines with or without 5-Aza treatment were determined through qRT-PCR.


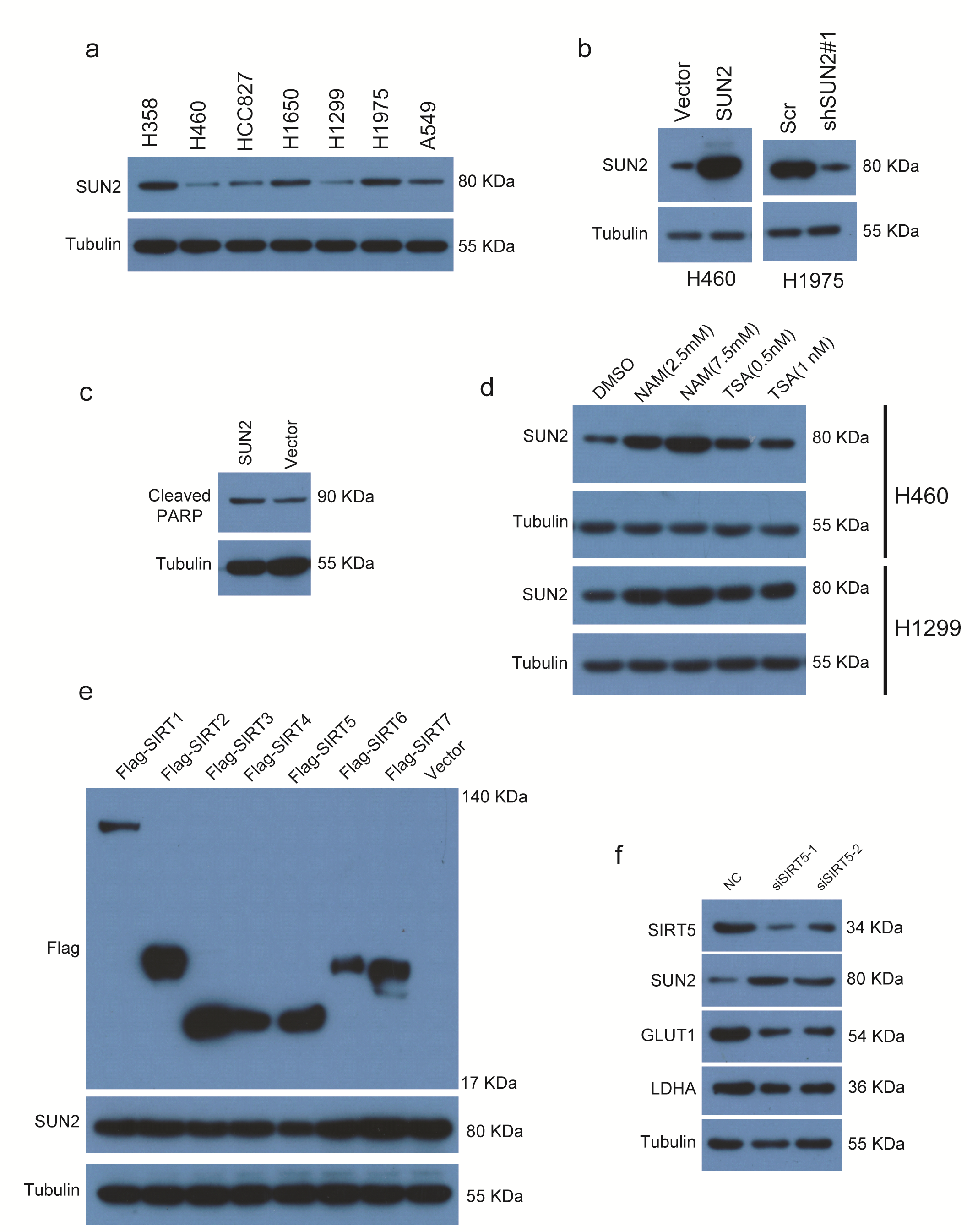


**Supplementary Figure 9.** **Full length blots of data shown in main figures.** (**a**) The gels were initially cut ranged from 45 KDa to 100 KDa. (**b**) The gels were initially cut ranged from 45 KDa to 110 KDa (left) and 45 KDa to 100 KDa (right). (**c**) The gels were initially cut ranged from 45 KDa to 110 KDa. (**d**) The gels were initially cut ranged from 45 KDa to 110 KDa. (**e**) The gels were initially cut ranged from 17 KDa to 140 KDa. (**f**) The gels were initially cut ranged from 25 KDa to 100 KDa.
